# Flow cytometry based profiling of leukocytes: A new method for diagnosis of tropical theileriosis in crossbred cattle

**DOI:** 10.14202/vetworld.2015.1379-1385

**Published:** 2015-12-11

**Authors:** Ramesh B. Jagtap, Amit Gupta, Sushama R. Chaphalkar

**Affiliations:** 1 Department of Virology and Immunology, Vidya Pratishthan’s School of Biotechnology, Baramati, Pune, Maharashtra, India; 2 Director, Vidya Pratishthan’s School of Biotechnology, Baramati, Pune, Maharashtra, India

**Keywords:** clinical examination, flow cytometry, hematology, lymphocytosis, *Theileria annulata*

## Abstract

**Aim:**

In India, dairy industries are important for the livelihood of small scale farmers and dairy owners. Tropical theileriosis, mostly affecting dairy cattle and buffaloes is a major threat to dairy and related industries. Tropical theileriosis is caused by *Theileria annulata*, a hemoprotozoan parasite transmitted by Ixodid ticks of *Hyalomma* spp. In the present study, we examined the clinical signs, hematological parameters and flow cytometric profile of whole blood in 30 theileriosis affected crossbred cattle. The aim of our study is to analyze, in comparison with clinical and hematological diagnosis, whether flow cytometry based profiling of leukocytes could be used as better, quick and alternative method for diagnosis and screening of bovine tropical theileriosis in crossbred cattle.

**Materials and Methods:**

In this study, we screened parasites in 30 peripheral blood samples from clinical cases of theileriosis by Giemsa’s staining technique in crossbred cattle. Hematological analysis was done to estimate hemoglobin (Hb) content, total red blood cell (RBC) count, total leukocyte count and differential leukocyte count. Further, flow cytometric analysis of whole blood was carried out to study leukocytes profile in affected cattle.

**Results:**

Microscopic examination of stained blood films revealed the presence of piroplasms in erythrocytes and schizonts in lymphocytes. Hematological examination revealed significant (p<0.05) decrease of Hb percent (Hb %), reduced total RBC and total leukocytes, lymphocytosis, eosinopenia, and neutropenia compared to that of apparently healthy cattle. Flow cytometric profiling of leukocytes revealed the severe effect on shape, size, and granularity of leukocytes, marked decrease in granulocytes and 3-5 fold increase in lymphocytes count compared to clinically healthy cattle. Thus, in both methods, namely conventional and flow cytometric analysis, marked lymphocytosis and decrease in other blood cell counts were observed compared to clinically healthy cattle.

**Conclusions:**

From results, it can be concluded that though conventional staining techniques and hematology are efficient in diagnosis of theileriosis, leukocytes profiling based on flow cytometry combined with clinical examination could be a quick, novel and alternative method for diagnosis and screening of clinical tropical theileriosis in crossbred cattle. Thus, there is potential to offer a flow cytometry based diagnostic service for tropical theileriosis in crossbred cattle.

## Introduction

Tropical theileriosis is a tick-borne hemoprotozoan disease caused by a small irregular shaped hemoprotozoon parasite, *Theileria annulata* which is transmitted through bite of ticks of *Hyalomma* spp. It resulted in severe economic losses in tropical and subtropical countries and also drastically affects the immune status of animals [1]. Among confirmed cases, incidence reported was varying between 31% and 45% in crossbred cattle. The predominant clinical sign in affected animals are enlargement of superficial prescapular and prefemoral lymph nodes near tick infested site, high fever, pale or yellowish mucous membrane, anorexia, drop in milk production, straw colored urine, black feces, anemia, emaciation, prostration, etc. [2-4]. In severe cases diarrhea [5] and even systemic signs like lateral recumbency was noted in certain cases [6]. After the bite of the tick, the sporozoites present in the gut are inoculated into the animal body. Infection thus initiated through the draining lymph node and sporozoites transform into schizonts in lymphocytes. Schizonts further transform into merozoites and infect erythrocytes of the host animal. This is followed by the development of piroplasms in erythrocytes which can be infective to ticks. The theilerial piroplasms in erythrocytes and schizonts stage in lymphocytes can be detected for diagnosis of disease.

Apart from the clinical symptoms, commonly used laboratory method for diagnosis is the detection of piroplasms in erythrocytes and Schizonts in lymphocytes by Giemsa’s or Leishman’s staining of thin blood films [1,4]. Further, diagnosis is supported by assessing the hematological parameters namely, hemoglobin percent (Hb %), total erythrocyte count (TEC), total leukocyte count (TLC), differential leukocyte count (DLC), packed cell volume, mean corpuscular Hb (MCH), MCH concentration, etc. The available methods *viz*. microscopic examination of stained blood films for detection of parasites, analysis of hematological parameters for supportive diagnosis, possess certain limitations and pitfalls. Rapid, reproducible, sensitive, high throughput method for diagnosis and screening of theileriosis from field samples in cattle is lacking.

Flow cytometry is a powerful tool for enumeration of lymphocyte subpopulations in peripheral blood, characterization of the composition of complex cell populations involved in clinical disorders, immunophenotyping [7], cross-matching of tissues in organ transplantation and characterization of lymphomas and leukemias, etc. In comparison with traditional methods, flow cytometry based assays are more rapid, quantitative, and precise and can be used for diagnosis of various clinical disorders. Flow cytometry has emerged as one of the new technologies in veterinary clinical laboratories for diagnosis of various disorders [7-11].

Thus, our study aimed to clinically examine suspected animals for theileriosis, its supportive diagnosis through microscopic examination of stained blood films, hematology and further to develop flow cytometry based assay for diagnosis and screening of suspected field samples for theileriosis in crossbred cattle.

## Materials and Methods

### Ethical approval

This work was approved by Institute Animal Ethical Committee (IAEC), under the component Animal disease Surveillance of “Biovillage” scheme of Vidya Pratishthan’s School of Biotechnology, Baramati, India.

### Clinical examination of suspected animals

The Holstein Friesian crossbred cattle aged between 3 and 5 years suspected for theileriosis were examined thoroughly for clinical signs and symptoms. The animals with high fever above 104°F are examined further whether there is enlargement of prescapular lymph nodes, color of feces and urine, breathing difficulties, feeding behavior, corneal opacity and emaciation.

### Sample collection

About 5 ml of blood in ethylenediaminetetraacetic acid (EDTA) coated tubes was collected from crossbred cattle suspected for theileriosis from Baramati area of western Maharashtra, India. A total of 40 blood samples from lactating cattle aged between 3 and 6 years were collected and used for hematological and flow cytometric analysis. Blood smears were prepared from fresh blood immediately after collection. Crossbred cows were grouped into an infected group (n=30) showing clinical signs *viz*. high fever, enlarged peripheral lymph nodes, ≥4% parasitemia by microscopic examination and clinically healthy crossbred cows (n=10) with no visible clinical signs and no parasitemia in erythrocytes or lymphocytes by microscopic examination of stained blood films.

### Hematological examination

The TEC was performed by a manual method using hemocytometer and diluting fluid as saline. The TLC was performed using improved Neubauer hemocytometer and white blood cells diluting fluid. Cyanomet Hb method was used for the estimation of Hb (g/dl). Thin blood smears were prepared from the fresh peripheral blood and stained by Giemsa’s stain. Further, DLC was performed by manual method as per Feldman *et al*. [12].

### Flow cytometric analysis

**Figure-1:**
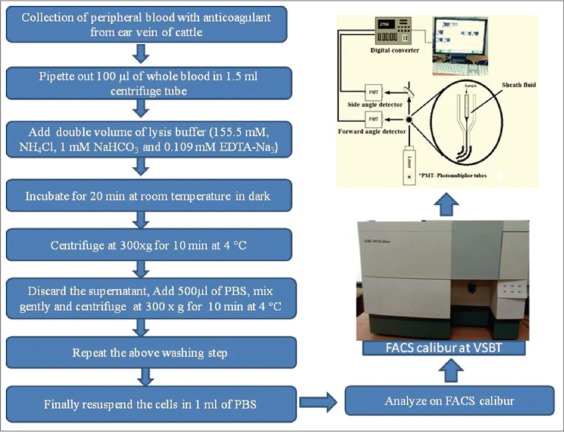
Flow chart of flow cytometry based profiling of leukocytes from whole blood of cattle.

Blood (5 ml) samples from clinically healthy and theileriosis suspected cattle were collected in EDTA-coated vacutainers. To study the differential profiling of leukocytes, briefly, 100 µl of whole blood of clinically healthy as well as infected cattle was lysed with lysis buffer (155.5 mM, NH_4_Cl, 1 mM NaHCO_3_ and 0.109 mM EDTA-Na_3_). After addition of lysis, buffer samples were incubated for 15 min at room temperature in the dark. The samples were then centrifuged at ×300 g for 15 min at 4°C, and the cells washed two times with phosphate-buffered saline (PBS) and finally resuspended in 1 ml of PBS. The normal as well as infected samples of theileriosis were kept in ice cold and dark during the whole procedure as well as during storage before analysis. The flow cytometric samples were analyzed within 24 h in an FACS calibur flow cytometer (Becton Dickinson). The flow cytometer was equipped with a 488 nm argon laser and Cell Quest TM Software (Becton Dickinson). 10, 000 events were collected from each sample. The leukocyte subpopulations, monocytes and granulocytes, were identified by their forward as well as side scatter characteristics, enclosed in electronic gates, and separately analyzed for fluorescence intensity. The results were calculated as the percent gated cells of 10,000 events. A brief flow chart of flow cytometry based profiling of leukocytes in whole blood of cattle was showed in Figure-1.

### Statistical analysis

To determine the statistical significance among different variables, means of hematological parameters were compared using SPSS program (Statistical Program for Social Sciences) version 17. One-way ANOVA was used for comparison between two groups (clinically healthy control and diseased cattle). All hematological values were expressed as mean and standard error of mean and p<0.05 was considered as statistically significant.

## Results and Discussion

In India, rearing of crossbred cattle is economically beneficial and important to farmers because of its high milk production capacity than indigenous cattle that is depicted in the recent share of milk production of crossbred cattle which is more than half of total cattle milk production [13]. The major disadvantage in rearing crossbred and exotic cows is poor adaptability to environmental and feeding conditions than indigenous breeds and thus crossbred cattle could be more prone to diseases especially hemoprotozoan diseases (theileriosis) because of the presence of more exotic blood [14]. Our group focused on mostly HF cross, or Jersey crossbred animals reared in Baramati area of Maharashtra, India. In the past few decades, cases of bovine tropical theileriosis were reported frequently in India [15-17], especially in Maharashtra [18]. Preventive measures such as the development of vaccines against theileriosis in India were reported previously [19,20]. The currently available licensed vaccine for theileriosis in India is Rakshavac-T (Indian Immunologicals Pvt. ltd.) which is recommended for prophylactic vaccination against *T. annulata* in crossbred and exotic cattle. Though treatment with oxytetracycline and buparvaquone were curative in most instances, still better curative and preventive measures are needed for the control of the disease. In field conditions diagnosis of theileriosis is carried out by submitting the peripheral blood samples collected at height of temperature to nearby veterinary diagnostic laboratory and detection of parasites in Giemsa or Leishman stained blood smear. Further, diagnosis is supported by analysis of hematological parameters in suspected cases. Our group focused on clinical examination of suspected cases, detection of parasites in Giemsa stained blood films, hematological analysis and further flow cytometric analysis of whole blood to profile leukocytes compared to clinically healthy cattle.

**Figure-2:**
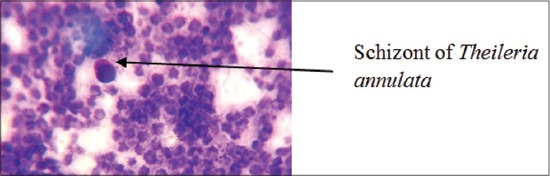
Schizonts of *Theileria annulata* in lymphocytes of infected cattle (Giemsa stain; oil immersion).

The clinical examination (Table-1) showed that most of the animals were with high fever (100%). Enlargement of prescapular and poplitial lymph nodes (90.00%), anorexia in few cases, labored breathing in 60% of cases, red-coffee colored urine and even dark feces were noted in few cases. Anorexia was less prominent in most of the cases, as the most of cows slowly fed than healthy cows. In certain previous reports [21] of clinical examination of theileriosis infected cattle showed similar but less severe signs, i.e., 18.51% cases of swelling of lymph nodes and hematuria, but anorexia reported was higher (42.62% cases), pale mucous membrane in the most of cases (42.59%) and conjunctivitis in few cases (29.62%). Similar findings were reported by Sandhu *et al*. [22] and *Radostits et al*. [23]. Anorexia could be attributed to persistent fever and in the early stage of the disease hyperplasia of lymphoid tissues could led to the enlargement of superficial lymph nodes. The corneal opacity may be the result of white blood cells infiltration. As anemia is one of the pathognomic signs of the disease, decreased TEC and hemoglobin concentration was observed in infected cattle compared to clinically healthy cattle. Microscopic examination of stained blood films revealed the presence of piroplasms in erythrocytes (86%) (Figure-2) and Schizont’s in lymphocytes (73.33%). However, Al-Emarah *et al*. [24] reported 69.43% and 25.43% erythrocyte as well as lymphocytic stage of *T. annulata* parasites while both stages in 5.26% cases in naturally infected cattle. Erythrocytic and lymphocytic stages of parasites in cattle, buffaloes, and horses were reported previously with variation in the percentage of occurrence [25-28].

**Table-1:** Clinical and microscopic findings among theileriosis suspected crossbred cattle.

Clinical sign	Infected cattle	

	Total number (30)	Percentage
Fever (above 104°F)	30	100
Enlargement of prescapular and prefemoral lymph nodes	27	90.00
Anorexia	18	60.00
Pale mucous membrane	20	66.67
Respiratory signs	18	60.00
Straw reddish urine	12	40.00
Emaciation	18	60.00
Corneal opacity	4	13.33
Dark feces	10	33.33
Piroplasms in RBC’s	26	86.67
Schizont’s in WBC’s	22	73.33

RBC=Red blood cells, WBC=White blood cell

Hematological examination (Table-2) revealed that there is significant decrease of Hb to an average of 5.84±0.85 g/dl, TLC (10^3^/μl) was decreased to 5.56±0.58 and TEC (10^6^/μl) was decreased to 5.24±0.65 compared to clinically healthy cattle. DLC revealed eosinopenia, lymphocytosis (83% of cases) associated with Neutropenia and 10% of cases (Table-2) showed lymphocytopenia associated with Neutrophilia and insignificant increase in monocytes count in few cases, in which slight decreased lymphocyte count was observed. There is slight variation compared to previous reports of theileriosis, in which mostly lymphocytopenia is reported [18,26,29] but lymphocytosis was also reported previously by certain researchers [4]. It was observed that in neutropenia cases; there is mostly increase in lymphocyte count and vice versa in remaining cases. Hence, neutropenia and lymphocytosis observed could be relative in those instances.

**Table-2:** Hematological parameters in normal and infected cattle.

Parameters	Clinically healthy cattle (n=10)	Clinical theileriosis (n=30)
RBCs (10^6^/μl)	7.91±1.03^a^	5.24±0.65^b^
Hb (g/dl)	11.96±3.23^a^	5.84±0.85^b^
TLC (10^3^/μl)	8.10±0.22^a^	5.56±0.58^b^
Neutrophils (10^3^/μl)	3.26±0.43^a^	2.14±0.26^b^
Lymphocytes (10^3^/μl)	5.02±0.26^a^	7.085±0.64^b^
Eosinophils (10^3^/μl)	0.42±0.034^a^	0.31±0.009^b^
Basophils (10^3^/μl)	0.023±0.006	0.018±0.006
Monocytes (10^3^/μl)	0.321±0.016	0.350±0.022

Means bearing different superscripts differ significantly along column at p<0.05, one-way ANOVA, RBC=Red blood cells, Hb=Hemoglobin, TLC=Total leukocyte count

Flow cytometry is generally used for elucidating the structural and functional properties *viz*. shape, size, granularity, etc. of the cell suspended in a stream of fluid as they pass through laser light either one, two or three. Further, the flow cytometer is used for differential enumeration of peripheral blood cells, cross-matching of tissues in organ transplantation and characterization of lymphomas and leukemias, immunophenotyping, i.e., detection of cell surface markers *viz*. cluster of differentiation; cell cycle analysis for apoptosis of cells, viability studies, estimation of total proteins, enzyme activity, gene expression analysis, etc., [30,31]. Number, shape, size and granularity of the cells in the fluid are estimated through forward and side scatter of the instrument. Light scatter properties reveals that there is a direct relationship between forward scattered light and cell volume, and this property of light is utilized in flow cytometry. The scattering of light i.e., coherent light source (488 nm, blue) using forward scatter (small angle scattering between 0.5°C and 5°C) measures shape and size of the cell and side scatter (large angle scattering between 15°C and 150°C dark field) measures complexity and granularity of the cell. The FACS can be used to measure the live cells and dead cells in the form of forward and side scatter; dead cells are more in side scatter and lower in forward scatter, whereas it is vice-versa for live cells [32-34].

**Figure-3:**
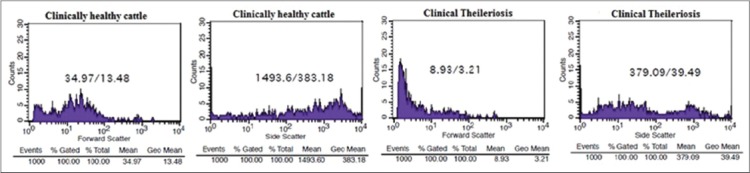
Flow cytometric analysis of clinically healthy and theileriosis infected crossbred cattle using forward (shape and size) and side scatter (granularity) to estimate the counts.

**Figure-4:**
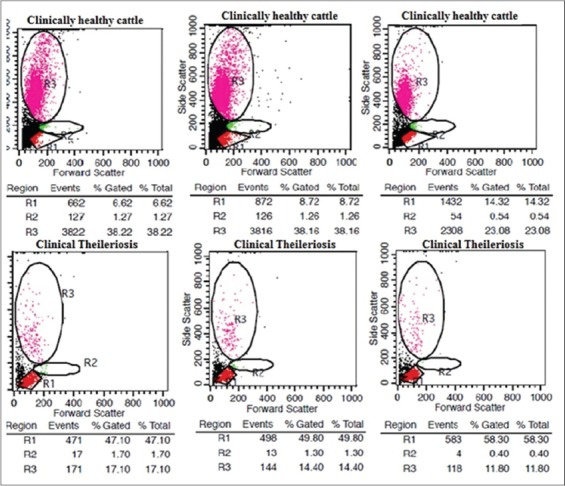
Flow cytometric analysis of clinically healthy and theileriosis infected crossbred cattle to estimate the lymphocytes, monocytes, and granulocytes count: Decrease in granulocyte count and marked lymphocytosis in infected cattle compared to clinically healthy cattle.

As showed in scatter diagram (Figure-3), the profiling of leukocytes by flow cytometer revealed that *T. annulata* affects severely shape, size and granularity of peripheral blood leukocytes. Granulocyte count was decreased sharply averaging 52.64±5.26% of 30 cases studied compared to clinically healthy cattle (Figure-4). Further, it was found that there was 3-5 fold increase in lymphocyte count in theileriosis suspected cases compared to clinically healthy crossbred cattle. Hematological and flow cytometric analysis showed a significant decrease in TLC (leukogram), eosinophils and neutrophils while lymphocytes showed a significant increase in comparison with clinically healthy control animals. The continuous harmful effects of toxic metabolites of *Theileria* spp. on the hematopoietic organs, especially bone marrow and their interference with the process of leucogenesis could be responsible for changes in leukocytes count. In response to entry or invasion of *Theileria* parasites, lymphocytes or monocytes count could show relative increased value. Lamia [35] reported similar findings in *Theileria* infected cattle. Significant neutropenia, decreased TLC and TEC could be due to breakdown of red blood cells by *Theileria* spp. or its toxic metabolites, which further could led to stimulation of the phagocytosis by lymphocytes and monocytes to remove fragmented and dead cell debris and toxic remnants of ruptured red blood cells. Which further increase the tissue demand of neutrophils that led to a reduction of neutrophils in peripheral blood circulation. Similar findings *viz*. red cell destruction, decreased TEC, TLC were reported previously in cattle [2,36].

## Conclusions

Thus, this study revealed that there is preliminary evidence of the prevalence of bovine tropical theileriosis in Baramati taluka of western Maharashtra. The diagnosis and screening of bovine tropical theileriosis can be done with conventional staining techniques and hematology. However, leukocytes profiling based on flow cytometry combined with clinical examination could be a quick, novel and alternative tool for diagnosis and screening of clinical tropical theileriosis in crossbred cattle and thus, there is potential to offer flow cytometry based diagnostic service for tropical theileriosis in crossbred cattle. Further studies could be done to develop more sensitive and accurate flow cytometric assay for other hemoprotozoan diseases *viz*. babesiosis, anaplasmosis in cattle and small ruminants.

## Authors’ Contributions

The study was designed, and protocols were carried out by RBJ. Sample collection as Veterinarian and further manuscript was written by RBJ. The FACS machine was operated by AG and overall approval by director, SRC. All authors have read and approved the final version of the manuscript.
